# Diagnostic disclosure, communication quality, and patient experience in autosomal dominant polycystic kidney disease

**DOI:** 10.1093/ckj/sfag351

**Published:** 2026-10-06

**Authors:** Malik Djadda, Fadi Haidar, Patrick Guirchoun, Sandra Sarthou-Lawton, Sylvie Coscoy, Dominique Joly

**Affiliations:** Service de Néphrologie, Hôpital Bichat and Université Paris Cité, Paris, France; Service de Néphrologie, Hôpitaux universitaires de Genève, Genève, Switzerland; Association PolyKystose France (PKD France), Saclay, France; Association pour l’Information et la Recherche sur les maladies Rénales Génétiques (AIRG-France), BP 78, cedex 06, Paris, France; Laboratoire Physico-Chimie Curie, Institut Curie, PSL Research University, CNRS UMR 168, Sorbonne Université, Paris, France; Service de Néphrologie, Hôpital Necker-Enfants Malades and Université Paris Cité, Paris, France; F-CRIN INI-CRCT (Cardiovascular and Renal Clinical Trialists), Nancy, France

**Keywords:** autosomal dominant polycystic kidney disease, diagnosis, disclosure, nephrology referral, patient-reported experience

## Abstract

**Background:**

In autosomal dominant polycystic kidney disease (ADPKD), the age and circumstances of diagnosis, the quality of initial disclosure, and how patients experience it have not been systematically described.

**Methods:**

Cross-sectional web-based survey in France (2019–2024) of adults with self-reported ADPKD recruited via patient organizations. Participants reported diagnostic context, who first disclosed the diagnosis, perceived information quality, and rated tact and overall experience on 0–5 scales. Primary outcomes were poor tact and very negative diagnostic experience (scores 0–1). Multivariable models included prespecified covariates tailored to each outcome.

**Results:**

Among 1021 respondents, median age at diagnosis was 26 (IQR 18–36) years. Diagnosis followed family screening (40%), symptom-driven work-up (27%), or incidental findings (21%), and occurred earlier in women and after parent-initiated screening. Initial disclosure was most often by radiologists (49%) rather than nephrologists (22%). Poor tact, reported by 25%, was associated with radiologist versus nephrologist disclosure [adjusted odds ratios (aOR) 2.50, 95% confidence interval (CI) 1.57–3.98]. Very negative experience, reported by 29%, was associated with poor tact (aOR 4.55, 95% CI 2.90–7.12) and poor information (aOR 2.52, 95% CI 1.53–4.15). Delayed or absent nephrology follow-up was reported by 40% and was associated with reporting no post-disclosure resolution (aOR 4.34, 95% CI 3.10–6.09).

**Conclusions:**

ADPKD was often disclosed outside nephrology, and patients frequently reported poor tact, inadequate information, and very negative diagnostic experiences. These findings identify communication quality and timely nephrology follow-up as potential targets for improving the ADPKD diagnostic experience.

## Graphical Abstract

**Graphical Abstract:**
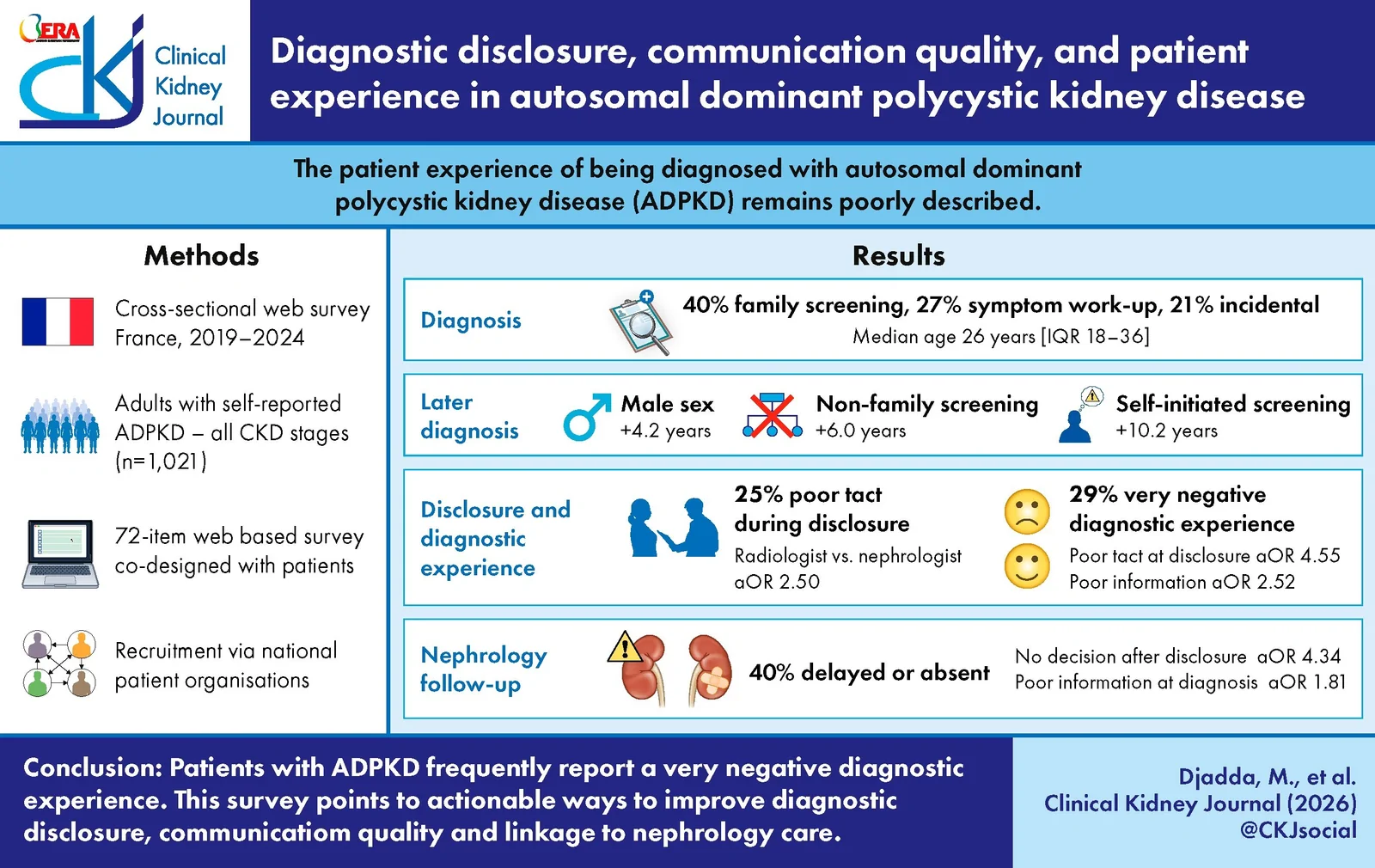

KEY LEARNING POINTS **What was known:**

- Diagnostic disclosure and early linkage to nephrology and genetic counseling are emphasized in ADPKD care recommendations, but patient-reported real-world disclosure experiences are incompletely described.

**This study adds:**

- Age at diagnosis varied substantially by diagnostic pathway, sex, and screening initiator; self-initiated family screening occurred ∼10 years later than parent-initiated screening.
- Initial disclosure was most often made by radiologists within imaging-based pathways; poor tact was more frequently reported after radiologist than nephrologist disclosure.
- Very negative experience was associated with poor tact and poor information, while delayed nephrology follow-up remained common, although less frequent in recent diagnostic eras.

**Potential impact:**

- These findings provide large-scale patient-reported evidence on ADPKD diagnostic disclosure and identify communication quality and linkage to nephrology care as potential areas for future intervention.

## INTRODUCTION

Autosomal dominant polycystic kidney disease (ADPKD) is the most common inherited kidney disease, characterized by progressive cyst development leading to kidney failure [1]. Beyond the somatic burden of pain, organ enlargement, and extrarenal manifestations, ADPKD carries distinct psychosocial weight. Its hereditary nature imposes unique anxieties regarding disease transmission, family planning, and identity [2, 3].

For many at-risk individuals, ADPKD remains an observed disease in relatives until a personal diagnosis marks a salient transition into the disease. As established in oncology, the quality of diagnostic disclosure is a key component of the patient experience. Better-managed disclosure has been associated with greater trust and shared decision-making, whereas poorer disclosure has been linked to greater anxiety and feelings of guilt [4].

Diagnosis typically occurs via family screening, symptom evaluation, or increasingly, as an incidental finding. The optimal timing of diagnosis remains ethically complex: while early identification allows for monitoring and lifestyle interventions, it may impose the psychosocial burden of a chronic label without immediate curative options—a “sword of Damocles” effect described in qualitative studies [5]. This tension is intensifying with advances in imaging and prenatal testing.

The 2025 KDIGO guideline emphasizes that diagnosis should be accompanied by counseling on prognosis and genetics [6]. However, the real-world implementation of these recommendations depends on how, where, and by whom the diagnosis is communicated. Evidence describing routine diagnostic pathways and the patient experience of disclosure remains limited.

We conducted the PK-DIAG survey to capture the diagnostic pathway from the patient perspective. We hypothesized that modifiable features of disclosure are associated with adverse patient experiences and subsequent engagement with care. Accordingly, the main objectives of this study were to (i) describe diagnostic pathways and disclosure practices, (ii) characterize the patient experience of diagnosis, and (iii) identify factors associated with poor disclosure and downstream engagement with nephrology care.

## MATERIALS AND METHODS

Study design and population

PK-DIAG was a cross-sectional, web-based survey conducted in France (February 2019–August 2024) among adults (≥18 years) with self-reported ADPKD across chronic kidney disease (CKD) stages (including dialysis and transplant), disseminated via national patient organizations.

Questionnaire development

A 72-item questionnaire was developed from literature review and expert consensus, refined through pilot testing, reviewed by patient associations [Association PolyKystose France (PKD France) and AIRG-France (Association pour l’Information et la Recherche sur les Maladies Rénales Génétiques)], and digitally pre-tested for usability (*n* = 41).

Patient and public engagement summary

PKD France and AIRG-France reviewed questionnaire wording, supported pilot testing, and disseminated the survey through mailing lists and social media. Findings are being shared with patient groups to inform educational materials on diagnostic disclosure and nephrology referral.

Measures

Domains included diagnostic circumstances, disclosure experience, perceived information quality at disclosure, health status, and health-related quality of life [Autosomal Dominant Polycystic Kidney Disease Impact Scale (ADPKD-IS), a validated disease-specific instrument with a 14-day recall period; higher scores indicate greater impact].

Disclosure experience and information

Because no ADPKD-specific validated instrument exists for routine disclosure quality, tact and overall diagnostic experience were each assessed with a single-item numeric rating scale (0 = worst possible, 5 = best possible); exact item wording and anchor labels are provided in Table S6. Perceived information was categorized as sufficient versus insufficient, clear versus unclear, and accurate versus inaccurate; “poor information” denotes any negative rating.

Outcomes

Primary outcomes were poor tact and very negative diagnostic experience, each measured on a study-specific single-item 0–5 scale (0 = worst; 5 = best). For primary analyses, scores were dichotomized a priori as 0–1 versus 2–5 to identify clearly negative experiences and facilitate interpretation. Full score distributions and floor/ceiling effects are reported in Table S7. Poor tact was also analyzed using an alternative 0–2 versus 3–5 threshold (Table S9).

Data collection and ethics

Participation was voluntary and anonymous; no directly identifiable data were collected, in compliance with the European General Data Protection Regulation (GDPR). The study was approved by the AP-HP Centre IRB (CERAPHP.5, IRB #00011928).

Deduplication

Duplicate/triplicate entries were removed using a prespecified algorithm (pseudonym-based grouping and internal consistency checks) followed by manual review.

Statistical analysis

We used descriptive statistics, multivariable linear regression with HC3 robust standard errors for age at diagnosis, and logistic regression for binary outcomes. Covariates were prespecified separately for each model according to clinical plausibility, prior literature, and temporal relevance. The poor-tact model included age at diagnosis, sex, education, diagnostic context, and first discloser; full model specifications are provided in the corresponding table footnotes. Analyses used complete cases and report the analytical *n*. We described the full 0–5 distributions of tact and experience scores (Table S7), estimated diagnostic year from survey year and reported ages, and summarized key outcomes by diagnostic era (Table S8). Variables measured at survey time (current mood and ADPKD-IS) were included only as exploratory adjustments in the very-negative-experience model. Analyses were performed in R version 4.3.1.

## RESULTS

### Participant characteristics

After removing 32 duplicate or triplicate entries, 1021 unique responses were included in the final analysis (Table 1). Mean age at the time of the survey was 51.2 ± 14.0 years (*N* = 797), and 531/797 (67%) were women. Among respondents with education data (*N* = 895), 742/895 (83%) reported at least a bachelor’s degree. Kidney failure had been reached in 28% of respondents (7% on dialysis, 21% transplanted). A family history of ADPKD was reported by 639/811 (79%); among respondents with a family history who answered the index-case item (*N* = 634), 88/634 (14%) identified themselves as the first diagnosed individual in their family. Item-level missingness was present; therefore, denominators vary across variables and are reported throughout the text and tables. Geographically, respondents were distributed across metropolitan France; the density of participation by department is illustrated in Fig. 1.

**Table 1:** Participant characteristics. Overall cohort (*N* = 1021); *N* are denominators, values are *n* (%) unless stated.

Sociodemographics	
Age, years (mean ± SD)	51.2 ± 14.0
Female sex	531 (66.6%)
*Education level (N = 895)*	
Lower than bachelor’s degree	153 (17.1%)
Bachelor’s degree	349 (39.0%)
Master’s degree or higher	393 (43.9%)
*Employment status (N = 900)*	
Employed (full-time or part-time)	547 (60.8%)
Retired	214 (23.8%)
Sick leave/disability	70 (7.8%)
Other (student, unemployed, homemaker)	69 (7.7%)
**Clinical characteristics**	
*Kidney replacement therapy (KRT) status (N = 837)*	
Not on KRT (CKD G1-G5)	605 (72.3%)
Dialysis (current or prior)	60 (7.2%)
Kidney transplant (current or prior)	172 (20.5%)
*Kidney function of non-KRT participants (N = 464)*	
eGFR, ml/min/1.73 m² (mean ± SD)	54.4 ± 32.1
CKD Stage 1–2 (eGFR ≥ 60)	188 (40.5%)
CKD Stage 3 (eGFR 30–59)	158 (34.1%)
CKD Stage 4–5 (eGFR < 30)	118 (25.4%)
Self-reported hypertension	549 (68.5%)
Self-reported liver cysts (any)	615 (62.4%)

Participant characteristics (n = 1021). Values are mean ± SD or n (%), with percentages calculated among respondents with available data for each variable. Denominators vary because of item-level missingness and conditional questions. KRT, kidney replacement therapy; CKD, chronic kidney disease; eGFR, estimated glomerular filtration rate.

### Diagnostic pathways and age at diagnosis

Diagnostic pathways are summarized in Fig. 1 and Table 2. Diagnosis occurred most frequently via family screening (40%), followed by symptom investigation (27%) and incidental findings (21%). The median age at diagnosis was 26 (IQR 18–36) years; 18% were diagnosed before age 18, while prenatal or at-birth diagnosis was uncommon (1.4%).

**Figure 1:**
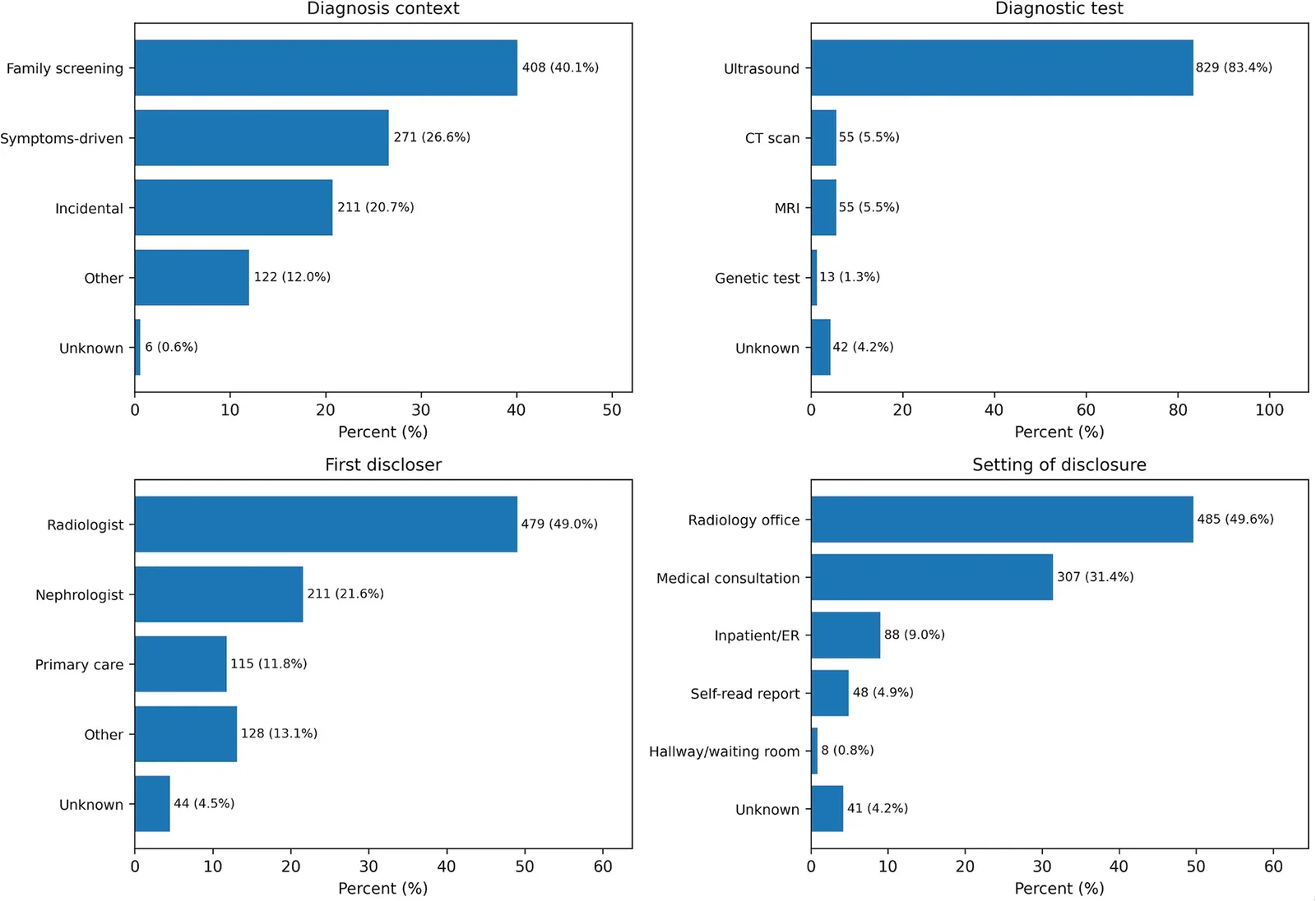
Diagnostic pathway overview. The diagrams summarize patient-reported diagnostic context (family screening, symptom-driven workup, incidental detection, other), main diagnostic test(s), first professional disclosing the diagnosis, and setting of disclosure. Values are *n*/*N* (%) based on available responses; denominators vary because of missing and conditional questions. CT, computed tomography; MRI, magnetic resonance imaging; ER, emergency room.

**Table 2:** Diagnostic pathways and disclosure experience. Overall cohort (*N* = 1 021); *N* are denominators, values are *n* (%) unless stated.

Diagnostic circumstances	Value
**Age at diagnosis, years (mean ± SD) (*N* = 1001)**	28.8 ± 13.4
**Early diagnosis (<18 years), (*N* = 1001)**	182 (18%)
**Context of diagnosis, (*N* = 1018)**	
Family screening (asymptomatic)	408 (40%)
Investigation of symptoms	271 (27%)
Incidental finding	211 (21%)
Other	122 (12%)
Unknown	6 (0.6%)
**Diagnostic modality, (*N* = 994)**	
Ultrasound	829 (83%)
CT scan/MRI	110 (11%)
Genetic testing	13 (1 %)
Unknown	42 (4 %)
**The disclosure encounter**	
**First professional to disclose, (*N* = 977)**	
Radiologist	479 (49 %)
Nephrologist	211 (22%)
Primary care physician	115 (12%)
Other specialist	128 (13%)
Unknown	44 (5%)
**Setting of disclosure, (*N* = 977)**	
Radiology office	485 (50%)
Medical consultation	307 (31%)
Inpatient/Emergency room	88 (9%)
Self-read report	48 (5%)
Hallway/Waiting room	8 (1%)
Unknown	41 (4%)
**Patient-Reported Experience (PREMs)**	
Tact score (0–5 scale), mean ± SD (*N* = 928)	2.6 ± 1.6
Poor Tact reported (Score 0–1), *n* (%) (*N* = 928)	229 (25%)
Overall experience score (0–5 scale), mean ± SD (*N* = 927)	2.4 ± 1.5
Very negative experience (Score 0–1), *n* (%) (*N* = 927)	268 (29%)
Information rated as insufficient/unclear/inaccurate, (*N* = 974)	218 (22%)
Immediate psychological support: unmet need, (*N* = 974)	394 (40 %)

Values are *n*/*N* (%) unless otherwise stated. Percentages are calculated on valid responses. Tact score and global experience score are single-item numeric rating scales ranging from 0 (worst possible experience) to 5 (best possible experience). Poor tact is defined a priori as a tact score 0–1; very negative experience as a global experience score of 0–1. “Poor information” is defined as information rated insufficient and/or unclear and/or inaccurate at the time of disclosure.

Age at diagnosis varied substantially by pathway. In multivariable linear regression (complete cases, *n* = 770), male sex, non-family-screening diagnostic contexts, and absence of a family history were each independently associated with older age at diagnosis (Fig. 2; Table S1). Men were diagnosed 4.2 years later than women (95% CI 2.26–6.17; *P* < 0.001), and symptom-driven or incidental diagnosis occurred ∼6 years later than family screening (both *P* < 0.001). A reported family history was associated with earlier diagnosis (−5.29 years; 95% CI −7.71 to −2.88; *P* < 0.001).

**Figure 2:**
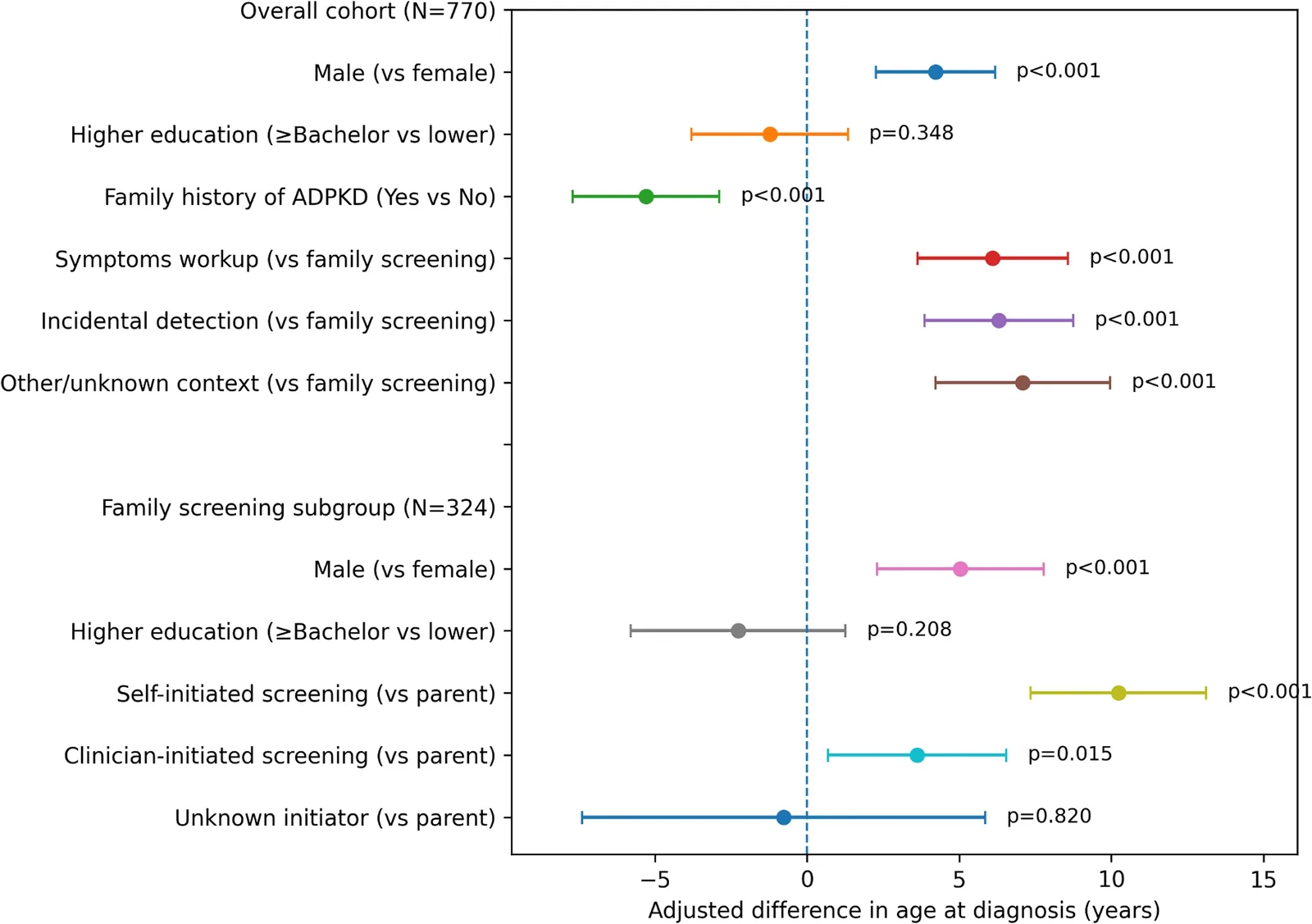
Multivariable factors associated with postnatal age at ADPKD diagnosis (linear regression). Points represent regression coefficients (*β*) and whiskers represent 95% confidence intervals. Coefficients reflect the adjusted mean difference in age at diagnosis (years) associated with each covariate. Upper panel: overall cohort model; lower panel: family-screening subgroup including screening initiator. Models were fitted as complete-case analyses using robust standard errors.

Within the family screening subgroup, the initiator of screening was a strong determinant of diagnostic timing. Parents initiated screening in 35% of cases, respondents themselves in 34%; parent-initiated screening was associated with a median age at diagnosis of 18 years versus 29 years for self-initiated screening (+10.2 years in multivariable analysis, 95% CI 7.35–13.11; *P* < 0.001). Women in the family screening cohort were also diagnosed earlier than men (median 20 vs. 23 years; *P* = 0.025).

### The disclosure experience

Ultrasound was the principal diagnostic modality (83%). Diagnosis was first disclosed by a radiologist in 49% of cases, a proportion that remained stable in those diagnosed in 2015–2024 (51%; Table S8). Disclosure took place in a radiology office in 50% of cases, during a medical consultation in 31%, and in an inpatient or emergency setting in 9% (Table 2). One quarter of respondents (25%) reported poor tact during disclosure (tact score 0–1 on a 0–5 scale; Table 2; Table S7).

Poor tact was associated with radiologist versus nephrologist disclosure (aOR 2.50, 95% CI 1.57–3.98), incidental versus family-screening diagnosis (aOR 2.11, 95% CI 1.36–3.27), and symptom-driven versus family-screening diagnosis (aOR 1.62, 95% CI 1.04–2.52) (Fig. 3; Table S2). The radiologist association persisted after adjustment for the broad diagnostic-context categories captured in the survey. Results were similar using the alternative poor-tact threshold (Table S9).

**Figure 3:**
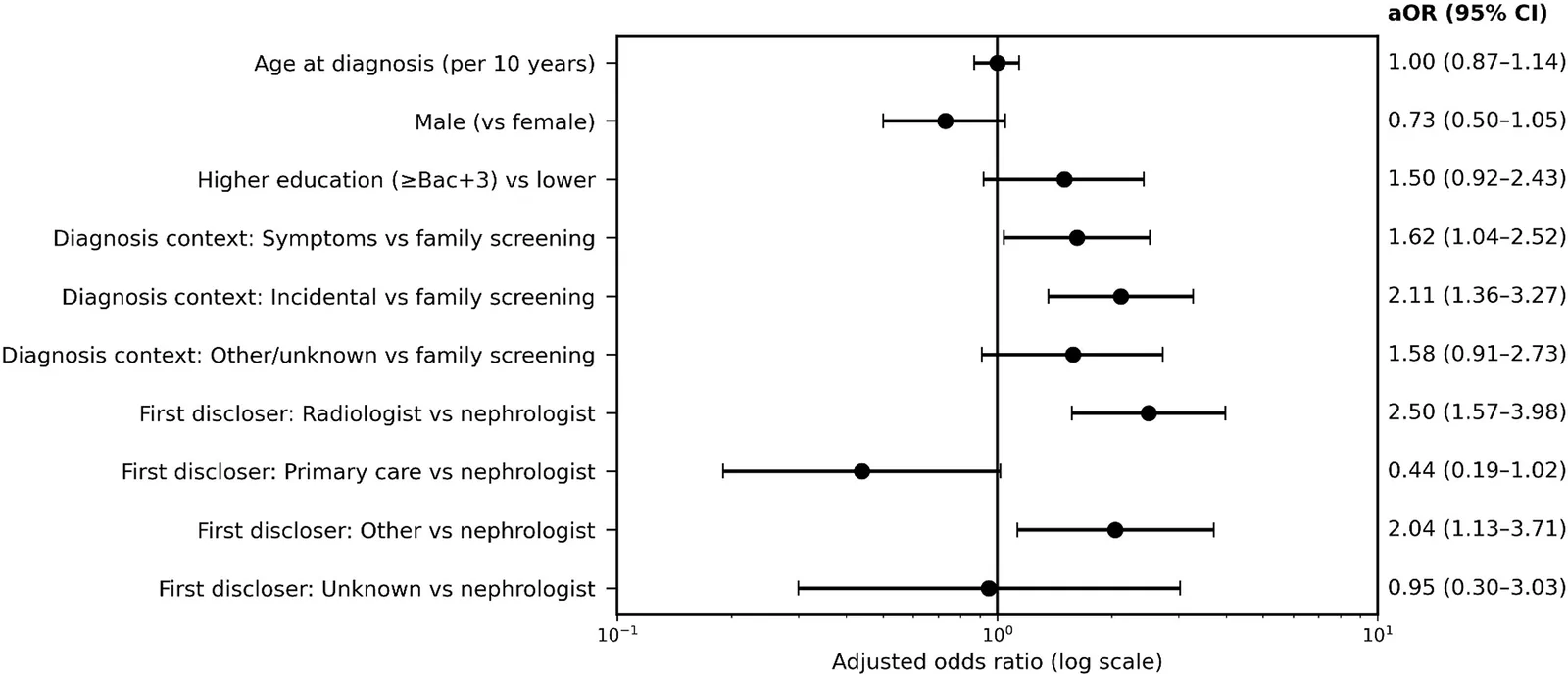
Multivariable factors associated with poor tact at ADPKD diagnosis (logistic regression). Forest plot of adjusted odds ratios (aORs) with 95% confidence intervals for poor tact (tact score 0–1 on a 0–5 scale). Points and whiskers (black) indicate aORs and 95% confidence intervals; numeric aOR (95% CI) values are displayed on the plot. Model fitted as a complete-case analysis (*n* = 803). Reference categories are indicated in the figure.

### Emotional response: **fears and feelings**

The most common fears at diagnosis were kidney failure (61%) and transmission to children (48%) (Table S3). In the hours and days following disclosure, respondents most commonly reported uncertainty (31%), indifference (30%), anxiety or stress (28%), shock (21%), and sadness or depression (14%) (Table S4).

A composite distress outcome (anxiety/stress and/or sadness/depression) was present in 36% and was independently associated with poor tact (aOR 1.93, 95% CI 1.29–2.90), poor information (aOR 1.99, 95% CI 1.30–3.04), older age at diagnosis (aOR 1.29 per 10-year increase, 95% CI 1.12–1.49), and greater worry about family history (aOR 1.52 per point, 95% CI 1.28–1.81) (Table S4).

### Overall diagnostic experience

Mean overall experience was 2.4 ± 1.5 on the 0–5 scale (Table 2; Table S7). A very negative experience (score 0–1) was reported by 29% overall and remained common among respondents diagnosed in 2015–2024 (40%; Table S8).

In multivariable logistic regression (*n* = 596), very negative experience was independently associated with poor tact (aOR 4.55, 95% CI 2.90–7.12), poor information (aOR 2.52, 95% CI 1.53–4.15), and greater worry about family history (aOR 1.37 per level, 95% CI 1.09–1.71) (Fig. 4; Table S5). Covariates measured at the time of the survey (including psychological support received and current mood) were included as exploratory adjustments and not interpreted as temporally antecedent to the disclosure experience.

**Figure 4:**
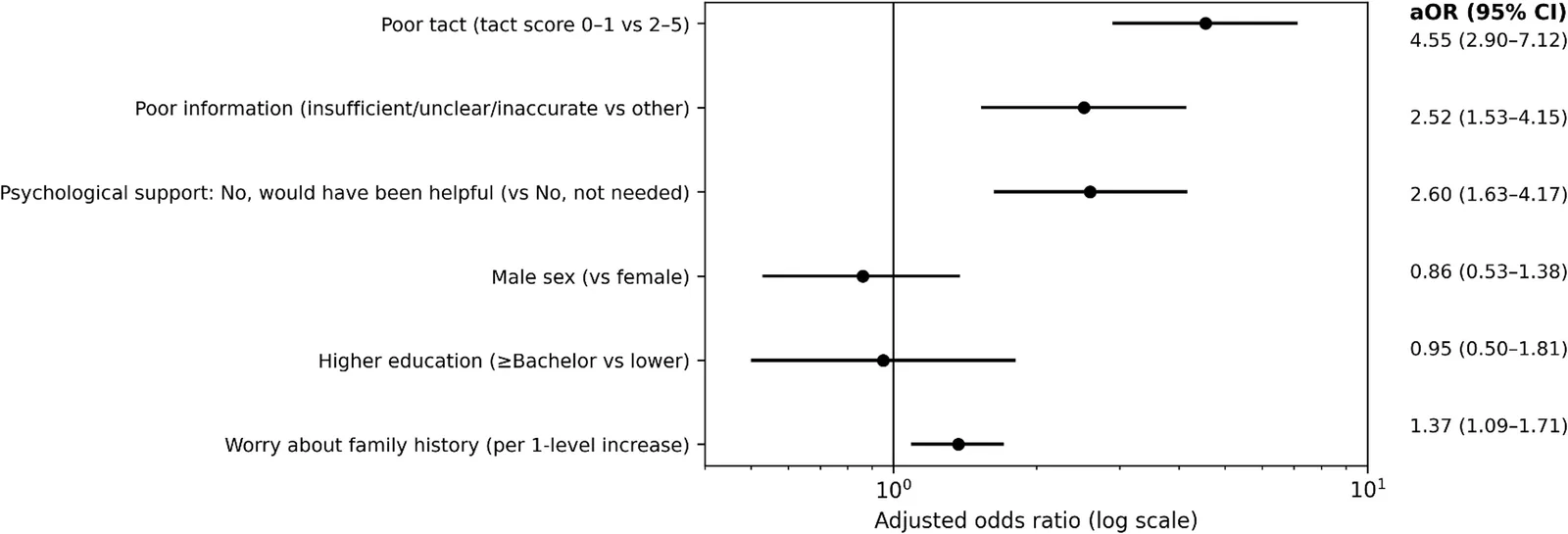
Multivariable factors associated with very negative diagnostic disclosure experience. Forest plot of adjusted odds ratios (aOR) from multivariable logistic regression for very negative experience, defined as a global diagnostic experience score 0–1 on a 0–5 scale (vs. 2–5). Points and whiskers indicate aORs and 95% confidence intervals; numeric aOR (95% CI) values are displayed on the plot. Model fitted as a complete-case analysis (*n* = 596). For clarity, this figure displays selected covariates with clear temporal anchoring at the time of disclosure; exploratory covariates measured at the time of the survey (current mood and ADPKD-IS) and the sparse category “psychological support received” are not displayed. Full model specification and estimates are provided in Table S5.

### Perceived timing

Two thirds of respondents (67%) felt their diagnosis had occurred at the right time, while 18% felt it was too early and 15% too late; both dissatisfied groups reported worse emotional quality of life and greater guilt-related impact (Table 3).

**Table 3:** Perceived timing of ADPKD diagnosis, preferred age to know one is affected within the family, and internal consistency analyses.

Panel A. Perceived timing of diagnosis and quality-of-life domains				
	Right time	Too early	Too late	*P* value
Perceived timing of diagnosis, *n*/*N* (%) (*N* = 960)	644 (67%)	174 (18%)	142 (15%)	-
ADPKD-IS Emotional domain [1–5], mean (SD)	1.9 (1.0)	2.6 (1.1)	2.4 (1.3)	<0.001
ADPKD-IS Guilt domain [1–5], mean (SD)	2.3 (1.4)	3.0 (1.5)	2.9 (1.5)	<0.001
Unmet need for psychological support at diagnosis, *n* (%)	192 (30)	110 (64)	82 (58)	<0.001
**Panel B. Preferred age to know one is affected by ADPKD within the family, by perceived timing of own diagnosis**				
**Preferred age category**	**Overall (*n* = 894)**	**Right time (*n* = 589)**	**Too early (*n* = 158)**	**Too late (*n* = 131)**
<18 years	287 (32)	207 (35)	16 (10)	64 (49)
18–30 years	388 (43)	271 (46)	66 (42)	47 (38)
>30 years	126 (14)	58 (10)	51 (32)	12 (9)
No opinion	93 (10)	53 (9)	25 (16)	8 (6)
Chi-square test *P* value < 0.001				
**Panel C. Discrepancy between actual and preferred age category, by perceived timing of own diagnosis (internal consistency)**				
**Discrepancy between actual and preferred age**	**Overall (*n* = 782)**	**Right time (*n* = 528)**	**Too early (*n* = 132)**	**Too late (*n* = 122)**
Earlier than preferred	104 (13)	42 (8)	61 (46)	1 (0.8)
Matched	356 (46)	278 (53)	49 (37)	29 (24)
Later than preferred	322 (41)	208 (39)	22 (17)	92 (75)

Chi-square test *P* value < 0.001.

(A) Association between perceived timing of diagnosis (classified as “too early,” “right time,” or “too late”) and patient-reported outcomes. Outcomes include the proportion of patients reporting an unmet need for immediate psychological support and scores for the ADPKD-IS Emotional and Guilt domains (presented as mean ± SD). Higher ADPKD-IS scores (range 0–5) indicate greater negative impact. (B) Preferred age category to know one is affected by ADPKD (<18 years, 18–30 years, >30 years, or no opinion), stratified by the participant’s retrospective appraisal of their own diagnostic timing. (C) Internal consistency analysis comparing the subjective appraisal of timing with the calculated discrepancy between actual and preferred age of diagnosis (categorized as earlier than preferred, matched, or later than preferred). Note: Values are *n* (%) unless otherwise indicated. Percentages in Panels B and C are column percentages. *P* values are derived from Chi-square tests.

When asked at what age it is most useful to know one is affected by ADPKD within the family, 43% favored 18–30 years, 33% favored before age 18, and 14% favored after age 30. Preferred age varied markedly by personal experience: those who felt diagnosed too early favored later testing, those who felt diagnosed too late favored earlier testing, and the discrepancy between actual and preferred age at diagnosis was internally consistent with these appraisals (Table 3).

### Post-diagnosis care

After disclosure, 39% of respondents reported a resolution to initiate specialist follow-up and 37% reported no specific resolution (multi-select; Table 4A). Among those with available follow-up data (*n* = 936), 60% reported prompt nephrology consultation, 38% a first consultation only years later, and 2% never or not yet; overall, 40% reported delayed or absent nephrology follow-up.

**Table 4:** Post-disclosure linkage to nephrology care and factors associated with delayed/never nephrology follow-up.

A. Timing of first nephrology consultation after diagnosis (*N* = 936)		
	*n*	%
Promptly (soon after diagnosis)	563	60
Delayed or never/not yet	373	40
Years later	355	38
Never/not yet	18	2
**B. Timing according to selected post-disclosure resolutions (multi-select)**		
Post disclosure resolution	Promptly *n* (row %)	Delayed/never^a^ *n* (row %)
Resolution to initiate specialist follow-up	302 (84)	59 (16)
No specific resolution	129 (37)	219 (63)
**C. Factors associated with delayed/never nephrology follow-up versus prompt follow-up (reference)**		
Predictor	Adjusted OR (95% CI)	P value
Resolution: no specific resolution	4.34 (3.10–6.09)	<0.001
Poor information quality at diagnosis	1.81 (1.23–2.68)	0.003
Age at diagnosis (per 10-year increase)	0.76 (0.65–0.88)	<0.001
Male sex (vs. female)	1.11 (0.79–1.56)	0.5
Higher education (≥Bachelor’s degree)	0.92 (0.58–1.45)	0.7
Diagnostic context: symptom-driven work-up (vs. family screening)	0.55 (0.36–0.84)	0.005
Diagnostic context: incidental finding/other/unknown (vs. family screening)	0.92 (0.63–1.35)	0.7

Panel A shows the timing of the first nephrology consultation after ADPKD diagnosis. Panel B displays the timing of nephrology follow-up according to selected post-disclosure resolutions (multi-select; categories not mutually exclusive). Panel C presents an exploratory multivariable logistic regression (complete cases, *n* = 774) examining factors associated with delayed or absent nephrology follow-up. ^a^Delayed/never follow-up combines “years later” and “never/not yet”. Poor information defined as information rated insufficient and/or unclear and/or inaccurate at diagnosis.

The concordance between post-disclosure resolutions and subsequent follow-up timing was marked: 84% of those who resolved to seek specialist care reported prompt follow-up, versus 37% among those with no specific resolution (Table 4B). Given the retrospective and multi-select nature of both items, this association should be interpreted descriptively, with caution regarding recall bias and reverse causality.

In exploratory multivariable analysis (*n* = 774), delayed or absent nephrology follow-up was independently associated with no specific post-disclosure resolution (aOR 4.34, 95% CI 3.10–6.09) and poor information at diagnosis (aOR 1.81, 95% CI 1.23–2.68). Older age at diagnosis was associated with lower odds of delayed follow-up (aOR 0.76 per 10 years, 95% CI 0.65–0.88), as was a symptom-driven versus family screening diagnostic context (aOR 0.55, 95% CI 0.36–0.84) (Table 4C).

## DISCUSSION

In this large patient-reported survey, ADPKD diagnosis was frequently first communicated by radiologists in imaging settings, a pattern that largely reflects imaging-based diagnostic pathways, and one quarter of respondents reported poor tact. Nearly one third described a very negative overall experience, strongly associated with the tact of disclosure and the perceived adequacy and clarity of information provided. Although the cross-sectional design precludes causal inference, these findings point to the disclosure encounter as an actionable target for quality improvement.

### Diagnostic pathways and family dynamics

Age at diagnosis varied substantially by pathway: diagnoses outside family screening occurred later, whereas parent-initiated screening occurred about a decade earlier than self-initiated screening, consistent with cascade-screening experience in inherited disorders [7, 8]. Men were diagnosed ∼4 years later than women. This is more plausibly related to diagnostic pathways than biology: women may have greater healthcare contact, including pregnancy-related imaging, and in our family-screening subgroup were diagnosed 3 years earlier than men. Broader sex differences in preventive healthcare and in the uptake of family screening, as documented in Lynch syndrome, may also contribute [9]. These findings support family-centered counseling that allows at-risk relatives to make informed decisions about whether and when to be tested.

### A young cohort

The median age at diagnosis in PK-DIAG (26 years) is younger than reported in population-based cohorts. In Olmsted County, mean age at diagnosis was 35 years among patients with a family history or genetic confirmation, and 56 years among those identified incidentally on imaging [10]. This most likely reflects the composition of our sample: 40% were diagnosed through family screening, 79% reported a family history, and recruitment through patient organizations probably favored families in which the disease is known and discussed. In populations with a higher share of incidental or symptomatic diagnoses, first disclosure by radiologists and the associated communication difficulties may be even more frequent than reported here.

### Early and presymptomatic testing: patient ambivalence and ethical considerations

A substantial minority perceived their diagnosis as occurring at the wrong time (too early or too late), and these groups reported worse emotional and guilt-related quality-of-life domains. This is consistent with qualitative work describing substantial emotional burden even in early stages [11] and with ambivalence toward presymptomatic testing [5]. Pediatric consensus statements similarly emphasize that testing in asymptomatic minors should not be routine and requires careful balancing of potential medical benefit and psychological harm [12]. In PK-DIAG, fears at diagnosis were dominated by the prospect of kidney failure and transmission to offspring, emphasizing disclosure encounters that address prognosis, inheritance risk, reproductive counseling, and psychosocial support.

### Disclosure practices and KDIGO guidance

KDIGO recommends counseling on prognosis, inheritance, family planning, and the benefits and harms of testing [6]. Imaging-based diagnosis may precede such counseling: ultrasound is first-line, and radiologists may be the first professionals able to name ADPKD after screening, symptom-driven, or incidental imaging. Our survey did not determine whether nephrology referral was already planned. The association between radiologist disclosure and poor tact persisted after adjustment for broad diagnostic context, but residual pathway differences remain possible. This finding therefore suggests a system-level handover gap rather than substandard radiology practice and supports prompt nephrology referral, standardized written information, and clear psychological-support pathways.

### Toward better-prepared diagnoses

Poorer tact after symptomatic or incidental diagnosis and the large difference between parent- and self-initiated screening support pre-test information for at-risk relatives. When the familial variant is known, targeted genetic testing may complement imaging and allow the timing and setting of disclosure to be planned; ultrasound in young adults may be inconclusive and require repeated assessment [6, 12]. Dedicated consultations, group education, or digital resources should be evaluated as ways to move the first ADPKD conversation upstream of testing.

### Psychosocial burden and patient priorities

The high prevalence of anxiety and fear of transmission in our cohort represents a significant burden, echoing findings on depression and psychosocial risk in ADPKD [13]. Recent patient-centered research by Mustafa *et al*. (EMPOWER-PKD) identified “psychological effect” as a leading priority for patients, ranking it higher than mortality in terms of daily impact [4]. Similarly, the SONG-PKD initiative confirmed that patients prioritize “life impacts”, such as anxiety and ability to work, well before the biological markers typically monitored by clinicians [14]. Our findings validate this prioritization and show that a negative diagnostic experience is strongly linked to *modifiable* factors (tact, information). The use of ADPKD-specific patient-reported outcome measures (PROMs), such as ADPKD-IS and ADPKD-PDS, can better capture these impacts that generic tools may underestimate [15–17].

### Bridging diagnosis and specialist care

Associations between immediate post-disclosure resolutions and subsequent nephrology follow-up (Table 4) should be interpreted cautiously: both may depend on the same underlying factors (referral pathways, disease severity at diagnosis, family context), and the alignment between a reported intention to seek specialist care and reported prompt follow-up may partly reflect consistency in self-reporting rather than a directional relationship. Nonetheless, the high proportion of respondents reporting delayed nephrology evaluation highlights a potential gap between initial disclosure and structured linkage to specialist care, supporting the need for clearer, standardized post-disclosure pathways.

### Patient organizations and patient-reported evidence

Patient associations were central to the design and dissemination of PK-DIAG, illustrating how community partnerships can generate actionable real-world evidence on diagnostic and care experiences. Unlike qualitative focus groups, which are highly informative on ethical and emotional dimensions but limited in scale [18, 19], this large patient-reported survey captures routine diagnostic practices across a broad sample and allows quantitative evaluation of the factors associated with adverse experiences [20, 21].

### Strengths and limitations

Strengths include the large patient-reported sample co-constructed with patient organizations, prespecified de-duplication with manual review, and multivariable analyses focused on encounter-level, directly actionable exposures.

Limitations include selection bias from web-based recruitment through patient organizations. Respondents were predominantly women, highly educated, and often had a family history. Recruitment through patient organizations may also have selected respondents who were more engaged with and informed about ADPKD, and who may have had higher expectations regarding communication or more readily recalled difficult disclosure experiences. Prevalence estimates may therefore not generalize to less-engaged patients, men, people with lower educational attainment, or those diagnosed incidentally. Diagnoses were self-reported and unverified, although recruitment was disease-specific and reported clinical characteristics were consistent with ADPKD. Disclosure circumstances and emotions were recalled retrospectively, sometimes decades later, and may have been reshaped by subsequent disease course; the composite distress outcome is particularly vulnerable. Nevertheless, core findings remained evident among respondents diagnosed in 2015–2024, and associations of very negative experience with tact and information persisted after adjustment for current mood and ADPKD-IS. Pregnancy-related diagnoses could not be isolated, and we did not know whether nephrology referral had been arranged at first disclosure. Tact and experience were study-specific single-item measures without full psychometric validation, although they used the full scale and the poor-tact findings were robust to an alternative threshold. Finally, the cross-sectional design precludes causal inference; associations between reported resolutions and follow-up may reflect reverse causality or reporting consistency.

## CONCLUSIONS

ADPKD was often disclosed outside nephrology; 25% reported poor tact, 29% a very negative experience, and 40% delayed or absent nephrology follow-up. Delayed follow-up became less frequent over time but was still reported by 23% of respondents diagnosed in 2015–2024. These findings identify communication quality and timely linkage to specialist care as potential targets for future interventions aiming to improve the diagnostic experience.

## Supplementary Material

sfag351_Supplemental_File

## Data Availability

De-identified participant data will be shared upon reasonable request to the corresponding author, subject to privacy and governance constraints.
