# Supplementary material for: Diagnostic disclosure, communication quality, and patient experience in autosomal dominant polycystic kidney disease
Source: Clin Kidney J. 2026 Oct 6;19(10):sfag351. doi: 10.1093/ckj/sfag351 (PMC13696891; doi:10.1093/ckj/sfag351)
Supplement: Supplementary file 1 [file sfag351_supplemental_file.docx]

***Supplementary material, PKDIAG survey***

[Supplementary Figure 1. Geographical distribution of PK-DIAG survey respondents.	2](#_Toc238044609)

[Supplementary Table 1. Multivariate linear regression analysis of factors associated with age at ADPKD diagnosis	3](#_Toc238044610)

[Supplementary Table 2. Multivariable logistic regression analysis of factors associated with poor tact diagnostic disclosure	4](#_Toc238044611)

[Supplementary Table 3. Fears expressed at the time of diagnosis (overall and by sex/family history).	5](#_Toc238044612)

[Supplementary Table 4. Feelings in the hours/days after diagnosis (multi-select) and factors associated with a composite distress outcome.	6](#_Toc238044613)

[Supplementary Table 5. Multivariable logistic regression analysis of factors associated with a very negative diagnostic disclosure experience.	7](#_Toc238044614)

[Supplementary Table 6. Survey instrument (key items)	8](#_Toc238044615)

[Supplementary Table 7. Distributional properties of the tact score and overall diagnostic experience score.	11](#_Toc238044616)

[Supplementary Table 8. Diagnostic contexts, disclosure setting, and key outcomes by estimated calendar year of diagnosis.	12](#_Toc238044617)

[Supplementary Table 9. Sensitivity analysis for the definition of poor tact in multivariable logistic regression (alternative threshold).	13](#_Toc238044618)

# Supplementary Figure 1. **Geographical distribution of PK-DIAG survey respondents.**

Bubble map showing the self-reported area of residence at the time of the survey, aggregated at the French department level to preserve confidentiality. Circles are centered on each department and scaled proportionally to the number of respondents reporting residence in that department (larger circles indicate higher counts). Department boundaries are shown for reference. Respondents living outside mainland France (overseas territories and/or outside France, depending on reporting) are summarized separately in the Supplementary material. The survey was administered in French; respondents were therefore French speaking.

# Supplementary Table 1. **Multivariate linear regression analysis of factors associated with age at ADPKD diagnosis**

Coefficients represent the adjusted difference in mean age at diagnosis (in years) compared to the reference group. The model was adjusted for sex, family history, diagnostic context, and education level. Robust standard errors (HC3) were used to account for heteroscedasticity. Complete-case analysis n = 770. CI = Confidence Interval; ADPKD = Autosomal Dominant Polycystic Kidney Disease.

| **Variable** | **Adjusted Difference in Age (Years)*** | **95% CI** | **P-value** |
| --- | --- | --- | --- |
| **Sex** |  |  |  |
| Female | Ref. | — | — |
| Male | + 4.22 | 2.26 to 6.17 | < 0.001 |
| **Family History of ADPKD** |  |  |  |
| No | Ref. | — | — |
| Yes | - 5.29 | -7.71 to -2.88 | < 0.001 |
| **Diagnostic Context** |  |  |  |
| Family Screening | Ref. | — | — |
| Symptoms / Workup | + 6.10 | 3.62 to 8.57 | < 0.001 |
| Incidental Finding | + 6.31 | 3.86 to 8.75 | < 0.001 |
| Other / Unknown | + 7.09 | 4.21 to 9.96 | < 0.001 |
| **Education Level** |  |  |  |
| Lower education | Ref. | — | — |
| Higher education | *NS* | *NS* | > 0.05 |

# Supplementary Table 2. **Multivariable logistic regression analysis of factors associated with poor tact diagnostic disclosure**

Adjusted odds ratios (aOR) with 95% confidence intervals for poor tact (tact score 0–1 on a 0–5 scale). Model fitted as a complete-case analysis (n=803). Abbreviations: aOR, adjusted odds ratio; CI, confidence interval; ADPKD-IS, ADPKD Impact Scale.

| **Variable** | **aOR** | **95% CI** |
| --- | --- | --- |
| Age at diagnosis (per 10 years) | 1.00 | 0.87-1.14 |
| Male (vs female) | 0.73 | 0.50-1.05 |
| Higher education (≥Bachelor’s degree vs lower) | 1.50 | 0.92-2.43 |
| **Diagnosis context (reference: family screening)** |  |  |
| Symptoms vs family screening | 1.62 | 1.04-2.52 |
| Incidental vs family screening | 2.11 | 1.36-3.27 |
| Other/unknown vs family screening | 1.58 | 0.91-2.73 |
| **First discloser (reference: nephrologist)** |  |  |
| Radiologist vs nephrologist | 2.50 | 1.57-3.98 |
| Primary care vs nephrologist | 0.44 | 0.19-1.02 |
| Other vs nephrologist | 2.04 | 1.13-3.71 |
| Unknown vs nephrologist | 0.95 | 0.30-3.03 |

# Supplementary Table 3. **Fears expressed at the time of diagnosis (overall and by sex/family history).**

| **Fear** | **Overall (n=442)** | **Men (n=114)** | **Women (n=255)** | **FH+ (n=290)** | **FH- (n=85)** |
| --- | --- | --- | --- | --- | --- |
| Dialysis or transplant | 271 (61.3%) | 82 (71.9%) | 154 (60.4%) | 177 (61.0%) | 59 (69.4%) |
| Transmission to children | 210 (47.5%) | 60 (52.6%) | 119 (46.7%) | 143 (49.3%) | 39 (45.9%) |
| Premature death | 85 (19.2%) | 20 (17.5%) | 53 (20.8%) | 56 (19.3%) | 18 (21.2%) |
| Medicalization / frequent visits | 63 (14.3%) | 18 (15.8%) | 36 (14.1%) | 48 (16.6%) | 7  (8.2%) |
| Pain | 49 (11.1%) | 15 (13.2%) | 26 (10.2%) | 29 (10.0%) | 13 (15.3%) |
| None | 59 (13.3%) | 13 (11.4%) | 36 (14.1%) | 40 (13.8%) | 9 (10.6%) |
| Other | 4  (0.9%) | 1  (0.9%) | 3  (1.2%) | 3  (1.0%) | 1  (1.2%) |

# Supplementary Table 4. **Feelings in the hours/days after diagnosis (multi-select) and factors associated with a composite distress outcome.**

| A) Feelings in the hours/days after diagnosis (multi-select) | | |
| --- | --- | --- |
| Feeling option | n | % |
| Uncertainty | 305 | 31.3 |
| Indifference | 292 | 30 |
| Anxiety/stress | 271 | 27.9 |
| Shock | 201 | 20.7 |
| Sadness/depression | 139 | 14.3 |
|  |  |  |
| B) Composite distress outcome | | |
|  | n | % |
| Distress feeling (anxiety/stress and/or sadness/depression) | 352 | 36.2 |
|  |  |  |
| C) Multivariable logistic regression for distress feeling | | |
| Predictor | OR (95% CI) | p-value |
| Intercept | 0.05 (0.02–0.12) | < 0.001 |
| Poor tact at disclosure (tact score 0–1) | 1.93 (1.29–2.90) | 0.002 |
| Poor information at diagnosis | 1.99 (1.30–3.04) | 0.001 |
| Male sex | 0.87 (0.59–1.28) | 0.49 |
| Diagnostic context: other (vs family screening) | 0.69 (0.39–1.23) | 0.21 |
| Diagnostic context: symptoms (vs family screening) | 0.97 (0.62–1.51) | 0.89 |
| Diagnostic context: incidental finding (vs family screening) | 0.54 (0.32–0.91) | 0.02 |
| Age at diagnosis (per 10-year increase) | 1.29 (1.12–1.49) | < 0.001 |
| Family history worry (per 1-point increase) | 1.52 (1.28–1.81) | < 0.001 |

# Supplementary Table 5. **Multivariable logistic regression analysis of factors associated with a very negative diagnostic disclosure experience.**

Complete-case model (N=596). Adjusted odds ratios (aOR) from multivariable logistic regression for very negative experience, defined as a global diagnostic experience score 0–1 on a 0–5 scale (vs 2–5). The model included poor tact, poor information (insufficient/unclear/inaccurate), psychological support at/just after diagnosis (Yes / No but would have been helpful / No not needed [reference]), male sex, higher education, current mood (1–10), ADPKD-IS overall score, and worry about family history (per level). Due to sparse data, the estimate for the “Yes, received” category (n=8) is not displayed in the table or main figure. Abbreviations: aOR, adjusted odds ratio; CI, confidence interval; ADPKD-IS, ADPKD Impact Scale.

| **Variable** | **Adjusted OR (95% CI)** | **P-value** |
| --- | --- | --- |
| **Experience of announcement** |  |  |
| Poor tact / lack of empathy | 4.55 (2.90–7.12) | < 0.001 |
| Poor information quality | 2.52 (1.53–4.15) | < 0.001 |
| **Psychological support** |  |  |
| No, not needed (Reference) | 1.00 (Ref.) | — |
| No, but would have been helpful | 2.60 (1.63–4.17) | < 0.001 |
| **Demographics & context** |  |  |
| Male sex (vs female) | 0.86 (0.53–1.38) | 0.53 |
| Higher education (≥Bachelor’s degree) | 0.95 (0.50–1.81) | 0.89 |
| Exploratory adjustment variables (measured at survey) |  |  |
| Mood score (per 1-point) | 0.98 (0.84–1.13) | 0.74 |
| ADPKD-IS overall score (per 1-point) | 0.94 (0.70–1.27) | 0.70 |
| Worry about family history (per level) | 1.37 (1.09–1.71) | 0.01 |

| Supplementary Table 6. Survey instrument (key items). The survey was administered in French. The table below details the exact wording of the questions and response options used to define the study variables, with their English translation. | | |
| --- | --- | --- |
| Variable Domain | **Question & Response Options (English Translation)** | **Original Wording (French)** |
| Diagnostic Context | **In what context was the diagnosis of ADPKD made?**  □ Family screening (asymptomatic)  □ Workup for symptoms (pain, hypertension...)  □ Incidental finding (check-up, surgery)  □ Other  □ Don't know | **Dans quel contexte le diagnostic de polykystose a-t-il été fait ?**  □ Dans le cadre d'un dépistage familial (je n'avais pas de symptôme)  □ Dans le bilan fait à cause de symptômes (douleurs, hypertension...)  □ Découverte fortuite à l'occasion d'un bilan de santé, d'une intervention chirurgicale  □ Autre  □ Je ne sais pas |
| Discloser Specialty | **Who was the first person to tell you that you had polycystic kidney disease?**  □ General Practitioner  □ Nephrologist  □ Radiologist  □ Don't know  □ Other | **Qui a été le premier à vous dire que vous aviez une polykystose ?**  □ Médecin traitant  □ Néphrologue  □ Radiologue  □ Je ne sais pas  □ Autre |
| Disclosure Setting | **In what context did you learn that you had polycystic kidney disease?**  □ Radiology office  □ Medical consultation office  □ Inpatient ward or Emergency Room  □ Hallway or waiting room  □ Self-read report  □ Don't know | **Dans quel contexte avez-vous appris que vous aviez une polykystose ?**  □ Dans le cabinet du radiologue ;  □ En consultation médicale ;  □ En hospitalisation ou aux urgences  □ Dans un couloir ou une salle d'attente  □ En lisant moi-même les résultats sur un compte-rendu  □ Je ne sais pas |
| Tact Score | **In your opinion, how was this diagnostic disclosure made to you?**  *(Numeric rating scale, integers 0–5)*  *0 = With no tact at all*  *5 = With great tact* | **Comment, selon vous, cette annonce diagnostique de polykystose vous a-t-elle été faite ?**  *(Echelle visuelle 0, 1, 2, 3, 4, 5)*  *0 = sans aucun tact*  *5 = avec un très grand tact* |
| Global Experience | **Overall, how did you experience this disclosure?**  *(Numeric rating scale, integers 0–5)*  0= extremely bad  5= very good | **Globalement, comment avez-vous vécu cette annonce ?**  *(Echelle visuelle 0, 1, 2, 3, 4, 5)*  0= extrêmement mal  5= très bien |
| Information Quality | **At the time your diagnosis was made, how do you judge the medical information provided to you?**  □ Impossible to say (did not seek info / too young)  □ Rather clear and understandable information  □ Rather insufficient, incomprehensible or inaccurate information | **Au moment de votre vie où le diagnostic de polykystose a été fait, comment jugez-vous les informations médicales qui vous ont été fournies ?**  □ Impossible à dire (à l'époque je n'ai pas cherché à avoir d'informations /ou j'étais trop jeune)  □ J'ai reçu à l'époque des informations plutôt claires et compréhensibles  □ J'ai reçu à l'époque des informations plutôt insuffisantes, incompréhensibles ou inexactes |
| Psychological Support | **At the time of diagnosis or immediately after, did you receive psychological support?**  □ Yes  □ No, I did not need it  □ No, it could have been useful but was not proposed | **Au moment du diagnostic de polykystose, ou dans les suites immédiates, avez-vous bénéficié d'une aide psychologique ?**  □ Oui  □ Non, je n'en ai pas eu besoin  □ Non, cela aurait pu être utile mais on ne me l'a pas proposé |
| Immediate Feelings | **What word(s) would best describe your feelings or state of mind in the hours or days following the diagnosis?**  □ Indifference / Carefreeness  □ Shock / Astonishment  □ Sadness / Depression  □ Anxiety / Stress  □ Uncertainty | **Quel(s) mot(s) décrirai(en)t le mieux vos sentiments ou votre état d'esprit dans les heures ou jours qui ont suivi le diagnostic de polykystose ?**  □ Indifférence / Insouciance  □ Choc / étonnement  □ Tristesse / dépression  □ Anxiété / Stress  □ Incertitude |
| Immediate Resolution | **After the disclosure of the diagnosis, what resolutions did you take?**  □ Change my lifestyle (drink more, stop smoking...)  □ Initiate specialist follow-up  □ Seek information on the disease by myself  □ None of the above | **Après l'annonce du diagnostic, quelles résolutions avez-vous pris ?**  □ Changer mon mode de vie (boire davantage, ne plus fumer, maigrir...)  □ Entreprendre un suivi spécialisé  □ Me renseigner sur la maladie par moi-même (internet)  □ Aucune des résolutions précédentes |
| Perceived Timing | **Overall, do you think your diagnosis of polycystic kidney disease was made...**  □ Too late (earlier diagnosis and care would have helped me)  □ At the right time  □ Too early (knowing early was rather negative for me) | **Vous pensez globalement que le diagnostic de votre polykystose a été fait ...**  □ Trop tard (un diagnostic et une prise en charge plus précoces m'auraient rendu service)  □ Au bon moment  □ Trop tôt (savoir précocement que j'avais cette maladie a été plutôt négatif pour moi) |
| Ideal Age | **At what age do you think it is useful to know if one is affected by polycystic kidney disease in your family?**  □ In childhood  □ In adolescence  □ Between 18 and 30 years old  □ After 30 years old  □ I have no opinion | **A quel âge vous semble-t-il utile de savoir si l'on est atteint de polykystose dans votre famille ?**  □ Dans l'enfance  □ Dans l'adolescence  □ Entre 18 et 30 ans  □ Après 30 ans  □ Je n'ai pas d'avis |

# Supplementary Table 7. **Distributional properties of the tact score and overall diagnostic experience score.**

The tact score and the overall diagnostic experience score were collected on single-item 0–5 numeric rating scales (0=worst, 5=best). We described their distributions (percentages by response option), median [IQR], and assessed floor/ceiling effects (percentage of responses at the minimum/maximum). In this cohort, both scores used the full response range, with moderate floor effects (tact: 17.2%; experience: 15.6%) and no marked ceiling effects (tact: 13.3%; experience: 11.3%). Across respondents with both scores available (n=916), tact and overall experience were moderately correlated (Spearman ρ=0.43, p<0.001), consistent with expected convergent validity. The 0–1 threshold was prespecified to capture the lowest two response categories, reflecting clearly negative disclosure experiences.

|  | Tact score(n=928) | Overall experience score(n=927) |
| --- | --- | --- |
| Distribution of scores, n (%) |  |  |
| Score 0 (worst) | 160 (17%) | 145 (16%) |
| Score 1 | 69 (7%) | 123 (13%) |
| Score 2 | 165 (18%) | 196 (21%) |
| Score 3 | 255 (28%) | 242 (26%) |
| Score 4 | 156 (17%) | 116 (13%) |
| Score 5 (best) | 123 (13%) | 105 (11%) |
| Summary statistics |  |  |
| Mean ± SD | 2.6 ± 1.6 | 2.4 ± 1.5 |
| Median [IQR] | 3 [2–4] | 2 [1–3] |
| Distribution characteristics |  |  |
| Floor effect (score=0) | 17% | 16% |
| Ceiling effect (score=5) | 13% | 11% |
| Worst two categories (scores 0–1) | 25% | 29% |
| Best two categories (scores 4–5) | 30% | 24% |

# **Supplementary Table 8**. Diagnostic contexts, disclosure setting, and key outcomes by estimated calendar year of diagnosis.

|  | ≤1999 | 2000–2009 | 2010–2014 | 2015–2024 |
| --- | --- | --- | --- | --- |
| Diagnostic context |  |  |  |  |
| Family screening | 184/415 (44.3%) | 70/179 (39.1%) | 33/87 (37.9%) | 36/98 (36.7%) |
| Symptoms work-up | 103/415 (24.8%) | 47/179 (26.3%) | 23/87 (26.4%) | 25/98 (25.5%) |
| Incidental | 71/415 (17.1%) | 46/179 (25.7%) | 19/87 (21.8%) | 24/98 (24.5%) |
| Other/unknown | 57/415 (13.7%) | 16/179 (8.9%) | 12/87 (13.8%) | 13/98 (13.3%) |
| Disclosure and outcomes |  |  |  |  |
| Radiologist disclosed diagnosis | 193/414 (46.6%) | 103/179 (57.5%) | 49/87 (56.3%) | 50/98 (51.0%) |
| Poor tact (0–1) | 105/408 (25.7%) | 51/177 (28.8%) | 20/86 (23.3%) | 24/98 (24.5%) |
| Poor tact (0–2) | 176/408 (43.1%) | 85/177 (48.0%) | 36/86 (41.9%) | 44/98 (44.9%) |
| Poor information | 82/412 (19.9%) | 45/179 (25.1%) | 17/87 (19.5%) | 30/97 (30.9%) |
| Very negative experience (0–1) | 104/407 (25.6%) | 52/173 (30.1%) | 30/87 (34.5%) | 39/98 (39.8%) |
| Delayed/never nephrology follow-up | 215/411 (52.3%) | 61/178 (34.3%) | 15/87 (17.2%) | 22/96 (22.9%) |

Footnote: Diagnostic era is based on an estimated calendar year of diagnosis, derived from survey year − (age at survey − age at diagnosis), and is therefore approximate. Only participants with non-missing age at survey and age at diagnosis contributed to these analyses; denominators vary by variable due to item-level missingness. Values are n/N (%).

# **Supplementary Table 9.** Sensitivity analysis for the definition of poor tact in multivariable logistic regression (alternative threshold).

| Predictor | Primary aOR (95% CI) Tact 0–1 vs 2–5 (n=803) | Sensitivity aOR (95% CI) Tact 0–2 vs 3–5 (n=777) |
| --- | --- | --- |
| Age at diagnosis (per 10 years) | 1.00 (0.87–1.14) | 0.96 (0.86–1.08) |
| Diagnosis context: incidental finding (vs family screening) | 2.11 (1.36–3.27) | 1.53 (1.02–2.29) |
| Diagnosis context: other/unknown (vs family screening) | 1.58 (0.91–2.73) | 1.26 (0.77–2.05) |
| Diagnosis context: symptoms work-up (vs family screening) | 1.62 (1.04–2.52) | 1.64 (1.11–2.41) |
| First discloser: other (vs nephrologist) | 2.04 (1.13–3.71) | 1.88 (1.12–3.16) |
| First discloser: primary care physician (vs nephrologist) | 0.44 (0.19–1.02) | 0.75 (0.42–1.34) |
| First discloser: radiologist (vs nephrologist) | 2.50 (1.57–3.98) | 2.58 (1.74–3.83) |
| First discloser: unknown (vs nephrologist) | 0.95 (0.30–3.03) | 1.63 (0.69–3.86) |
| Higher education (≥Bachelor’s degree vs lower) | 1.50 (0.92–2.43) | 1.36 (0.91–2.05) |
| Male sex (vs female) | 0.73 (0.50–1.05) | 0.87 (0.63–1.19) |

Footnote: Models adjusted for age at diagnosis (per 10 years), sex, education level (≥Bachelor’s degree vs lower), diagnostic context, and first discloser. The primary model is the main-text model (complete-case analysis, n=803; Supplementary Table 2 and Figure 3); the sensitivity model was fitted on n=777 complete cases.
